# Challenges experienced by Portuguese professionals in humanitude care for institutionalized elderly people during the pandemic

**DOI:** 10.1590/1980-220X-REEUSP-2021-0426

**Published:** 2022-01-05

**Authors:** Kátia Lilian Sedrez Celich, Rosa Cândida Carvalho Pereira de Melo, Mara Ambrosina de Oliveira Vargas, Francielly Zilli, Liliana Vanessa Lúcio Henriques, Jeane Barros de Souza

**Affiliations:** 1 Universidade Federal de Santa Catarina, Programa de Pós-Graduação em Enfermagem, Florianópolis, SC, Brazil.; 2 Escola Superior de Enfermagem de Coimbra, Coimbra, Portugal.; 3 Universidade Federal de Santa Catarina, Departamento de Enfermagem, Florianópolis, SC, Brazil.; 4 Universidade Federal da Fronteira Sul, Chapecó, SC, Brazil.

**Keywords:** Coronavirus Infections, Old Age Assistance, Humanization of Assistance, Institutionalization, Infecciones por Coronavirus, Asistencia a los Ancianos, Humanización de la Atención, Institucionalización, Infecções por Coronavírus, Assistência a Idosos, Humanização da Assistência, Institucionalização

## Abstract

**Objective:**

To understand the challenges experienced by Portuguese workers in humanitude care for institutionalized elderly people during the pandemic.

**Method:**

This is a qualitative study, supported by reflections on the Humanitude Care Methodology, carried out with workers from different areas in a nursing home for elderly people in Portugal. Data collection took place between September and October 2020, from individual and online interviews. The categorization proposed by Bardin was adopted as the analysis technique.

**Results:**

Three categories emerged: (1) self-protection and of the other with the subcategories fear of being contaminated and fear of contaminating the elderly; (2) maintenance of affective relationships, broken down into the subcategories absence of family members in the nursing home and personal protective equipment as a barrier to communication and approximation; and (3) confinement of the elderly who attended the Day Center, with the subcategories lack of family support/loneliness and change in the elderly’s routine.

**Conclusion:**

The main challenges experienced by Portuguese workers are related to the necessary changes in the performance of care practices due to the use of personal protection that was not used before, limitations in affective relationships, and restrictions in interaction spaces.

## RESUMO

**Objetivo:**

Compreender os desafios vivenciados por profissionais portugueses no cuidado em humanitude a idosos institucionalizados durante a pandemia.

**Método:**

estudo qualitativo, sustentado pelas reflexões da Metodologia do cuidado em Humanitude, realizado com profissionais de diversas áreas num residencial para idosos de Portugal. A coleta dos dados ocorreu entre setembro e outubro de 2020, a partir de entrevistas individuais e *online.* Adotou-se, como técnica de análise, a categorização proposta por Bardin.

**Resultados:**

emergiram três categorias: (1) proteção de si e do outro com as subcategorias medo de se contaminar e medo de contaminar o idoso; (2) manutenção das relações afetivas, desdobrada nas subcategorias ausência dos familiares no residencial e equipamento de proteção individual como barreira de comunicação e aproximação e (3) confinamento dos idosos que frequentavam o Centro de Dia, com as subcategorias falta de suporte familiar/solidão e alteração da rotina do idoso.

**Conclusão:**

os principais desafios vivenciados pelos profissionais portugueses estão relacionados às modificações necessárias no desempenho das práticas de cuidado devido a utilização de proteção pessoal antes não utilizada, limitação nas relações afetivas e restrições de espaços de convivência.

## RESUMEN

**Objetivo:**

Comprender los desafíos vividos por profesionales portugueses en el cuidado humanitud a ancianos institucionalizados durante la pandemia.

**Método:**

estudio cualitativo, basado en las reflexiones de la Metodología del cuidado Humanitud, realizado con profesionales de diversas áreas en una residencia para ancianos en Portugal. La recolección de datos ocurrió entre septiembre y octubre de 2020, tras entrevistas individuales y *online.* Se adoptó como técnica de análisis, la clasificación propuesta por Bardin.

**Resultados:**

surgieron tres clases: (1) protección de uno y de los demás con las subclases miedo de contaminarse y miedo de contaminar al anciano; (2) manutención de las relaciones afectivas, desdoblada en las subclases ausencia de los familiares en la residencia y equipo de protección individual como barrera de comunicación y aproximación y (3) confinamiento de los ancianos que frecuentaban el “Centro de Día”, con las subclases falta de soporte familiar/solitud; y alteración de la rutina del anciano.

**Conclusión:**

los principales desafíos vividos por los profesionales portugueses están relacionados a las modificaciones necesarias en el desempeño de las prácticas de cuidado debido a la utilización de equipo de protección antes no utilizada, limitación en las relaciones afectivas y restricciones de espacios de convivencia.

## INTRODUCTION

Coronavirus Disease 2019 (Covid-19) is the first pandemic of the 21st century, with a humanitarian cost that has surpassed 221,134,742 confirmed cases worldwide, resulting in 4,574,089 deaths by the beginning of September 2021. In Portugal, there are 1,047,710 registered cases, with 17,810 deaths^(1)^. After a year of pandemic, it should be noted that Covid-19 spread rapidly around the world, influencing the lives of billions of people. In particular, the elderly population is mentioned, as the aging process has made this public more vulnerable and susceptible to Covid-19, especially those living in Nursing Homes for the Elderly (*ERPIs* in Portugal)^(2)^.

In ERPIs, the elderly are cared for by health care workers, who have also suffered and are still intensely affected by the repercussions of the pandemic, being daily exposed to biological risks, work overload and psychological distress. Such workers experience psychological pressure in the face of the possibility of getting infected and spreading the disease to people under their care, generating anxiety, fear, depression, insomnia and anguish^(3)^.

The care for the elderly public has numerous action strategies favoring humanization and integrality in the care process. Among them, the Humanitude Care Methodology (HCM) is highlighted, which was developed in France, in the seventies, by Yves Gineste and Rosette Marescotti. HCM emerged from concerns about the way in which daily care for dependent and vulnerable people was developed, emphasizing dignity, freedom, and autonomy in care^(4)^.

Humanitude is an approach centered on the relations among people, between the caregiver and the individual to be cared for, involving a relationship in which both share their existence, in the construction of a balanced relationship^(4–5)^. The development of HCM is organized in a Structured Sequence of Humanitude Care Procedures (SSHCP), which is divided into five dynamic and successive stages.

The first stage, called pre-preliminary, represents the time to prepare the environment and the person to be cared for, avoiding surprising approaches, related to privacy, freedom, and autonomy. In the second stage, called preliminaries, an approximation with the person cared for is established through the gaze, word, and touch, always developed in this sequence. In the third stage, called sensory rebouclage, the coherence among sensory inputs (sight, hearing, touch, and smell) is observed, leading to a bodily sensation of well-being. In the fourth stage, emotional consolidation, the professional positively reinforces all the collaboration, efforts and progress achieved, valuing and thanking the person cared for at the time of the relationship. The last stage, reunion, is the moment when the continuity of care is affirmed and the next meeting is scheduled, reducing the feeling of abandonment^(5,6)^.

It should be noted that for the development of the five stages of the SSHCP, there are approximately 150 relational techniques, which need to be learned by professionals so that, during care, they can be intentionally performed^(5)^. Given the above, the guiding question of the study was raised: what are the challenges experienced by Portuguese professionals in humanitude care for the elderly living in an ERPI during the pandemic?

This investigation is warranted because it is a recent phenomenon, and the Covid-19 pandemic develops in real time, with long-term repercussions in the lives of institutionalized elderly people and especially in their caregivers’ lives, still with gaps in scientific evidence, lacking the watchful eye of researchers. Added to this, there are few studies addressing the care based on HCM in elderly residents of ERPI in times of pandemic. Based on this, the study aimed to understand the challenges experienced by Portuguese workers in humanitude care for elderly living in an ERPI during the pandemic.

## METHOD

### Design of Study

This is an exploratory study of qualitative approach^(7)^, supported by reflections on the Humanitude Care Methodology.

### Population

The team working in the nursing home consisted of 20 workers from different areas, such as nursing, social assistance, psychology, sociology, and occupational therapy.

### Local

The study was carried out in an ERPI in the North of Portugal, in which the HCM is already implemented and undergoing certification. In this nursing home, there are 28 elderly people, with other 30 elderly people attending the place only during the day.

### Selection Criteria

The inclusion criteria were professionals with training in HCM and experience in caring for the elderly in the ERPI during the Covid-19 pandemic. The exclusion criteria were being workers on vacation or on leave during the period of data collection. Of the 20 workers of the nursing home, eight met the inclusion criteria, and all were invited and accepted to participate in the study, with no refusals.

### Data Collection

Data collection took place during the months of September and October 2020, from individual, online interviews performed by the principal investigator, using *Zoom* platform. The interviews were recorded in digital voice audio recording, with an average duration of 50 minutes; then, they were transcribed by the principal investigator into a Microsoft Word document. Participants were invited with a formal invitation and interviews were scheduled in advance. The interview script consisted of sociodemographic data and a guiding question: what are the challenges experienced in humanitude care for the elderly in ERPI during the Covid-19 pandemic?

### Data Analysis and Treatment

The categorization proposed by content analysis was adopted as a technique and for data treatment^(8)^, which is a modality of analysis operationally consisting of three stages: pre-analysis, analysis, treatment of results, and interpretation. The study participants validated the interview content.

### Ethical Aspects

The ethical and legal principles postulated in Resolution No. 466/12 were followed^(9)^. The study was approved on July 18, 2020 by the Research Ethics Committee of a Federal University in southern Brazil, under protocol number 4.161.1725. Before conducting the interviews, the objectives of the study were presented and the Informed Consent Form (ICF) was sent to each participant, who signed the document and returned it via e-mail to the researchers. Participants were given access to the verbatim transcript obtained from the interviews and to the final study. As a guarantee of anonymity, the letter “E”, which means “entrevistado” (interviewee) was used to identify the study participants, followed by an Arabic number (E1, E2, E3, E4, E5, E6, E7, E8), according to the chronological order in which the interviews took place.

## RESULTS

Eight workers participated in the study, six women and two men. Ages ranged between 30 and 50 years, with most of them within 40 years. All participants had higher education qualifications in the following areas: three nurses, a social worker, a psychologist, a sociologist, an occupational therapist, and an administrator. Regarding the time working at ERPI, two workers had been working for six years and two for 23 years old, with the highest concentration being between eight and twelve years.

Content analysis produced three categories: (1) self-protection and of the other, in which two subcategories emerged: fear of being contaminated and fear of contaminating the elderly; (2) maintenance of affective relationships, where two subcategories were observed: absence of family members in the nursing home and personal protective equipment as a barrier to communication and approximation; and (3) confinement of the elderly who attended the Day Center, leading to two subcategories: lack of family support/loneliness and change in the elderly’s routine.

The category “Self-protection and of the other” is supported by two subcategories integrating the challenges workers experienced during the development of their attributions in humanitude care for elderly living in the ERPI in Portugal during the pandemic. In the first subcategory “Fear of being contaminated”, caregivers reported their fears and how they found, in HCM, a safe and effective way to continue caring in a humanized way:

*Although the entire team is very afraid of Covid, we are afraid of getting sick, afraid that our family members get sick (…) humanitude helped us (…) we knew that care for the elderly needed to be provided, and based on this affective relationship founded on the pillars and principles of humanitude, we knew that we would be able to keep everyone well (E3).*

*Even with all the fear we felt, we did not let ourselves be strayed from our path (…) decisions and practices remained in what is our focus, caring for the elderly in a humanized way (…) a path that it will never come back like the past, cares in force, never again! This is from the past (E4).*

The subcategory “Fear of contaminating the elderly” showed that workers were aware of the risk to which the elderly were exposed and wanted to use all resources to protect them:

*With the pandemic and all the limitation it brought, the activities had to go out of the routine we had, I have to be very careful to keep the elderly with some activity and at the same time maintain the necessary distance so as not to put them at risk (E5).*

*I decided to stay longer here at the nursing home, go home less frequently (…) I believe that this way I run less risk of bringing the virus inside and thus it is less likely that our elderly people will be infected (E7).*

The category “Maintenance of affective relationships” showed the complex reality for the preservation of the daily dynamics of fraternal bonds during the adverse conditions of the pandemic, as the elderly became vulnerable and susceptible to Covid-19, and it was necessary to find care strategies for these individuals’ physical and mental health protection. HCM was evidenced as an essential tool in this care. This category was supported by two subcategories, which subsidized the challenges the workers experienced. In the first subcategory “Absence of family members in the nursing home”, the participants reported the need for this interaction and how the presence of the family promotes good living for everyone at ERPI:

*We had a culture that was very open to family and friends, and with the Covid pandemic, we had to change that (…) the elderly had to experience physical distancing from their family members (E3).*

*We managed to align some situations to maintain proximity with the families, we used more technologies, more phone calls and video calls, more visits at the windows to try to alleviate this situation a little and allow the elderly and their families to see each other (E5).*

*I notice that there is a difference in their behavior, because they no longer have contact with family members, they no longer have their presence. Family members used to come to visit at any time and took them out and for walks, this was a huge break in their routine and in their conditions, even we miss it (E8).*

The subcategory “Personal protective equipment (PPE) as a barrier to communication and approximation” demonstrates the obstacle caused by the use of personal protective equipment in daily work and the significant impact that its use causes in the care of the elderly. Such feelings are exposed in the following speeches:

*Communication is more difficult because we have the mask, there are people who don’t hear that well and can’t follow our lips and don’t see our facial expression and this ends up limiting our approach and even our care (E8).*

*The contingency plan that was implemented, in terms of sanitary restrictions, has distanced us from each other (…) especially the use of mask and goggles, they have been a hindrance in our daily lives (E4).*

The category “Confinement of the elderly who attended the Day Center” is delineated by two subcategories expressing the restrictions and changes of several activities experienced by workers with regard to face-to-face social interactions, given the need for isolation of the elderly and different care required to prevent Covid-19 transmission. This fact is described in the subcategory “Change in the routine of care practices”, which can be observed in the following speech:

*We had 30 elderly people who came here every day and returned home only at the end of the day, and who were forced to stay at home when all this started (…) and this has affected us emotionally, because we know our role and our responsibilities (E5).*

Another challenge faced by workers in humanitude care is the concern for the elderly who experienced physical distancing and social and family isolation, which is characterized in the subcategory “Lack of family support/loneliness”, expressed in the statement:

*(…) we call constantly, especially in cases where there is less family support, many had to stay at home and not necessarily with their family, because they live alone and now they are even more alone, they report feeling sad and abandoned (E8).*

*(…) we continue to keep in touch with people at home for whom we realize, in fact, the big impact of breaking a routine that somehow organized them, along with a fear of what is next, it’s hard! We are also scared and we miss them all here with us, our home is not the same without them, the activities we used to do together were no longer done, we miss them and continuing here without them is difficult, very hard (E5).*

## DISCUSSION

The research findings point to different challenges related to the Covid-19 pandemic from the perspective of professionals working in the care of elderly people who live in an ERPI.

Study participants had different backgrounds, but all of them had been trained in HCM and were working in an ERPI context during the period of the COVID-19 pandemic. When asked about the challenges experienced in the care of institutionalized elderly in ERPIs during the COVID-19 pandemic, they identified several challenges they faced in this pandemic period.

The challenges experienced by workers in humanitude care for the elderly residing in an institution are related to the evident concern of self-contamination and the contamination of the elderly, as well as challenges resulting from the protection measures used to prevent this contamination. Thus, from the use of protective equipment to the disruption of family life within institutionalized elderly people or the impossibility of receiving the elderly at the Day Center, everything challenged workers to create strategies to minimize this distancing, with change in affective relationships and the confinement of the elderly being a challenge posed by the Covid-19 pandemic.

The pandemic has devastatingly affected those living in nursing homes, home residents who have the support of institutions, physicians and nurses, who had to deal with the rapid spread of the virus, modifying care strategies arising from the need to prevent the contagion^(10)^.

Participants identify fear as a huge challenge in care. Another study^(11)^ showed that health professionals, during the COVID-19 pandemic, showed stress and enormous psychological pressure due to the possibility of being contaminated and being able to contaminate the elderly they were caring for. However, here the participants reinforced that, despite the fear of being contaminated in the provision of care to the elderly and the fear of contaminating the elderly, the focus continued to be humanized care and for this HCM contributed positively with tools to manage the fear and anxiety caused by the pandemic.

HCM is based on the relationship between the caregiver and the person being cared for, allowing it to be operationalized through a Structured Sequence of Humanitude Care Procedures (SSHCP)^(4)^. Another study^(12)^ also highlights the importance of HCM in the management of basic negative emotions during the pandemic period, namely fear, anxiety, and despair, enhancing positive emotions generating more optimism, serenity, and confidence in everyone involved.

In times of Covid-19 pre-pandemic, family members distancing from the elderly was already pointed out as a generator of suffering for professionals working in long-stay institutions^(13)^. Thus, the absence of the family, due to confinement, was a huge challenge for care provision. During this period, the feeling of abandonment experienced by the elderly was difficult to manage, as some elderly did not understand the need for physical distancing from their family members^(12)^.

The participants of this study, recognizing the importance of the presence of the family for the elderly well-being, made use of technology, such as the use of video calls, in an attempt to compensate their absence. This premise was reinforced by a study^(14)^ stating that the use of technology seems to have mitigated the negative effect of the absence of the family during the period of confinement due to Covid-19.

We can understand, from the speeches presented, that this factor directly influences care practices of professionals who work following HCM. This distancing imposed by the pandemic is seen by workers as a challenge, because HCM qualifies family relationships and involvement in care practices, favoring open access for family members and thus strengthening interpersonal relationships^(6,15)^.

Moreover, the maintenance of direct affective relationships between the elderly and the workers is also affected by the Covid-19 pandemic due to the use of PPE. Due to the virus transmission characteristics, the recommendation regarding individual protection measures include, for example, the use of masks and face shields, which can hinder verbal and non-verbal communication between the workers and the elderly^(16)^.

The use of PPEs was registered by the participants in our study as a challenge when establishing communication with the elderly, recognizing the importance of the relation with maintenance of the relational pillars gaze, word, and expression of feelings and touch during care^(12,17)^. With HCM, caregivers understand that humanization of care, through the use of gradual relational techniques, related to gaze, word, and touch, favors assistance and interaction between the caregiver and the elderly^(15)^. Thus, in view of the Covid-19 pandemic, the maintenance of these pillars has become a challenge for health professionals who work in humanitude care and need to deal with the restrictions that prevent exposure of the face and touch.

Following this same line of need to prevent the spread of the virus, the impossibility of receiving elderly people cared at the Day Center raises new concerns for professionals accustomed to taking in a large number of elderly people in the institution.

The challenge of following the recommendations regarding social distancing and thus changing the care routine that relied on the provision of services at the Day Center led professionals to seek strategies capable of promoting multidimensional care for the elderly, even at a distance. The use of resources, such as calls to the elderly who live alone, can be understood as a strategy for maintaining the bond and reinventing modes of social interaction, seeking to maintain a connection between the health professional and the assisted elderly^(18)^.

However, even if professionals seek strategies to maintain the bond, we can understand this rupture in the routine of care provided by professionals to elderly users of the Day Care Center as a challenge experienced as it directly interferes in one of the steps and dynamics of care, which is that of emotional consolidation. This step is related to the consolidation of emotional memories awakened during the care relationship between the professional and the elderly, which will permeate the entire conduction of relational care practices^(6)^.

By not building a direct relationship with the elderly and, therefore, not being able to clearly carry out care practices in humanitude, seeking intermediary strategies to minimize this rupture, the distancing imposed by the Covid-19 pandemic is understood as a major challenge experienced by professionals in humanitude care. Thus, issues related to loneliness and social isolation can often be neglected by health and social services professionals, but the Covid-19 pandemic ended up exposing this aspect, which has led to a greater focus and commitment on the part of professionals in paying greater attention to social isolation and loneliness, particularly in the elderly, as they constitute the most vulnerable segment of the population^(19)^.

In this study, inclusion criteria were professionals trained in HCM, with experience in caring for the elderly in a nursing home and who worked during the pandemic. Therefore, as limitations of this study, we point out that the inclusion criteria restricted the number of participants and the selection of the institution.

## CONCLUSION

The Covid-19 pandemic changed the lives of different generations, influencing professional care practices in different areas of activity and levels of complexity around the world.

Faced with care practices guided by the Humanitude Care Methodology in Nursing Home Structures for Elderly People, Portuguese professionals highlight different challenges imposed by the pandemic. These challenges relate mainly to the changes necessary for the performance of care practices guided by a sequence of procedures based on the relationship between professionals and the elderly cared for. All these relationships were affected by Covid-19 and were challenges to the workers.

## Financial support

Coordenação de Aperfeiçoamento de Pessoal de Nível Superior (CAPES).

Programa de Excelência Acadêmica (PROEX) nº364/2021.
